# Estimation of Value-Based Price for Five High-Technology Medical Devices Approved by a Regional Health Technology Assessment Committee in Italy

**DOI:** 10.7759/cureus.24695

**Published:** 2022-05-03

**Authors:** Andrea Messori, Sabrina Trippoli

**Affiliations:** 1 Health Technology Assessment Unit, Regione Toscana, Firenze, ITA

**Keywords:** quality-adjusted life years, willingness-to-pay threshold, value-based price, cost-effectiveness, medical devices

## Abstract

Background and objectives

Value-based pricing (VBP) is used quite frequently for medicines, but its application to medical devices is very limited. The objective of the present study was to conduct a pilot experience of systematic estimation of the value-based price of medical devices from the perspective of our national health system. Our experience was focused on high-technology devices (class IIb/III and active implantable). The objective was to evaluate the applicability of VBP in a real-world setting and to estimate the value-based price of devices in all cases where this estimation was feasible.

Methods

The dataset analysed in this work consists of 24 new devices approved consecutively in the Tuscany region over the period from January 2020 to December 2021. Since the calculation of value-based price requires the availability of a cost-effectiveness analysis, we searched for this information for each of these devices. The Cost-Effectiveness Analysis (CEA) Registry of Tufts Medical Center (US) and the health technology assessment (HTA) reports of our region were considered adequate sources of these data. Standard equations of cost-effectiveness were applied to determine the value-based price for these devices, and these prices were compared with the corresponding real prices charged in our region.

Results

We found adequate information for five devices (21%) out of the total of 24. In three of these cases, the published analysis taken as a reference was based on Markov modelling. The comparison between value-based prices and real prices generally showed an acceptable concordance, though with a couple of outliers. An important finding is that, in a large proportion of cases (79%), the information needed for this calculation was lacking.

Conclusion

To our knowledge, this is the first experience in which an institution of the healthcare system has tried a systematic application of VBP in the field of high-technology devices. Our results are encouraging and suggest a wider application of cost-effectiveness in this field.

## Introduction

The concept of value-based pricing (VBP) has been debated in the scientific literature for a long time, and its application has generally been focused on medicines, particularly in the area of innovative agents [1-5]. A number of national regulatory agencies (e.g. in the UK, France, Germany, and Canada) have formally adopted algorithms that estimate the value-based price for newly introduced medicines [4,5]. Other countries systematically evaluate the cost-effectiveness ratio of new medicines, but the estimation of a value-based price is not mandatory in the pathway of regulatory approval and reimbursement [4]. In general, policies adopted by individual countries strongly depend on whether or not a willingness-to-pay (WTP) threshold has been formally recognised in the country concerned [6]. In cases where a formal recognition exists, the values of this threshold are similar among European countries, whereas more substantial differences can be found outside Europe. To our knowledge, no reports have described any systematic application of VBP in the field of medical devices. The current literature only offers sporadic applications of VPB to individual devices, particularly high-technology ones, which often reflect a research project sponsored by the manufacturer [7,8].

In Italy, the GRDM (Gruppo Regionale Permanente Sui Dispositivi Medici, Regione Toscana) is a regional working group that studies and manages high-technology medical devices on behalf of the Tuscany region. This group, which was formed in 2018, has a multidisciplinary composition. Its policy is that all health technology assessment (HTA) reports are published on the Tuscany region's website. As of April 2022, this website includes more than 120 HTA reports [9]. Documents produced by GRDM are subjected to further approval by the HTA regional committee, which converts recommendations issued by GRDM into binding regulations for the whole Tuscany region.

In a previous article [8], we analysed a group of high-technology devices that were consecutively approved in the Tuscany region from January 2020 to December 2021. This dataset represents a real-world experience conducted within the healthcare system of a European country and is specifically focused on the approval process of high-technology devices for in-hospital use. To our knowledge, other experiences of this type have not been reported in the scientific literature, so this dataset is particularly useful as a working example to test the methodology of VBP described herein.

In the present work, we describe a pilot experience in which we retrospectively applied the approach of VBP to these devices. Value-based prices were estimated according to standard methods of cost-effectiveness analysis (CEA) and compared with the current prices from the Italian market (“real prices”).

## Materials and methods

Study design

The dataset of Trippoli et al. [8] consisting of 24 high-technology devices has been the basis for the work described herein. Each of these 24 devices is associated with a rapid HTA report published on the Tuscany region's website [9], which has been the basis for the (favourable or unfavourable) decision made by the Tuscany region about purchasing the device. These 24 HTA reports are written in Italian. To apply the method of VBP, a cost-effectiveness model is needed in which both clinical effectiveness and costs are framed according to standard principles of HTA modelling. As inclusion criterion, we accepted such models from only two sources: (a) the CEA Registry [10], which is a worldwide compendium of cost-effectiveness reports managed for many years by the Tufts University in the United States; and (b) the above-mentioned regional HTA reports, provided that the cost-effectiveness model was judged to be appropriate and a specific reference to a peer-reviewed article was reported.

Estimation of value-based price

In comparing two hypothetical treatments denoted as A (novel treatment) and B (comparator or standard of care), the standard formula to determine the incremental cost-effectiveness ratio (ICER) is as follows:

$ICER = (cost_{A} - cost_{B}) / (QALYs_{A}- QALYs_{B})$ (Equation 1),

where QALYs are quality-adjusted life years.

In the estimation of value-based price for A, cost_A_ can firstly be split into the price of the device (price_A_) plus the other costs incurred in the clinical use of A (denoted as othercosts_A_):

$cost_{A} = price_{A}+ othercosts_{A}$ (Equation 2).

It should be noted that also cost_B_ includes the same two components (i.e. price_B _+ othercosts_B_); however, splitting cost_B_ is not mandatory if, as in the present case, the calculation is aimed at estimating the value-based price for A. Furthermore, it should be kept in mind that the original analyses frequently exclude both othercosts_A_ and othercosts_B_ when their two values are identical.

Since ICER is: $\small ICER = (price_{A} + othercosts_{A} - cost_{B})/gain_{QALYs}$ and incrementalcost_AvsB_ is: $\small incrementalcost_{AvsB} = price_{A} + othercosts_{A} - cost_{B}$, the equation of ICER can be re-written as follows:

$ICER = incrementalcost_{AvsB} / gain_{QALYs}$ (Equation 3),

where the difference QALYs_A_− QALYs_B_ has been denoted as gain_QALYs_.

Finally, the relationship between ICER and incrementalcost_AvsB_ is:

$incrementalcost_{AvsB} = ICER \times gain_{QALYs}$ (Equation 4).

Hence, $price_{A}+ othercosts_{A} - cost_{B} = ICER \times gain_{QALYs}$ (Equation 5), and $price_{A} = ICER \times gain_{QALYs} + cost_{B} - othercosts_{A}$ (Equation 6).

If one replaces ICER with the societal value of the WTP threshold (WTP_threshold_), the value-based price for A (denoted as valuebasedprice_A_) can be estimated as follows:

$valuebasedprice_{A} = WTP_{threshold} \times gain_{QALYs} - othercosts_{A} + cost_{B}$ (Equation 7).

This equation was used in our analysis. It should be kept in mind that the price based on this threshold of €60,000/QALY represents the highest acceptable price.

Finally, in secondary analysis, we also calculated the value-based prices according to a WTP threshold of €30,000/QALY. In this way, a range of prices can be proposed for each device. These additional price values derived from this second threshold, based on the lower threshold, are more likely to reflect the current trends in the market.

Selection of devices to be included in our analysis

A simple flowchart was employed to describe the process by which we handled our inclusion criterion. As regards the currency used in our analysis, our final results were expressed in euro; when necessary, different currencies were converted into euro by using the Oanda website (http://www.oanda.com/currency-converter/).

## Results

**Figure 1:**
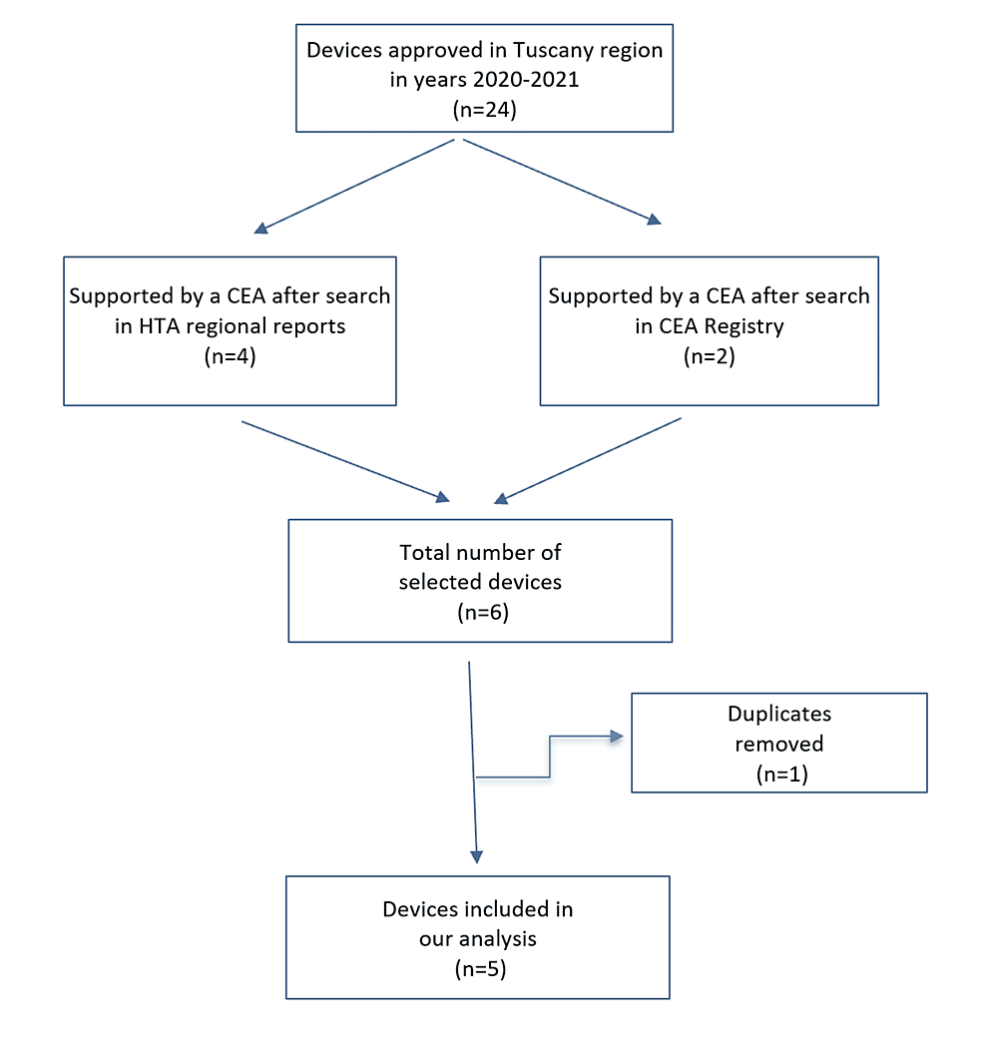
Flowchart of the process that selected the five devices studied in the present analysis. CEA, cost-effectiveness analysis; HTA, health technology assessment.

Among the 24 eligible devices, five (21%) were found to be supported by adequate cost-effectiveness data. The flowchart of this selection is illustrated in Figure 1.

Table 1 summarises the main characteristics of these five devices along with their sources of cost-effectiveness information. In three of these five cases, the published analysis taken as a reference consisted of a Markov model. All of these five devices are used in the cardiology setting; this finding seems to be casual even though it likely reflects the more frequent availability of CEAs in the cardiology setting compared with non-cardiologic ones.

**Table 1:** Information about the five devices included in the analysis and estimation of their value-based prices according to standard cost-effectiveness algorithms. Further details on these five devices can be found in the article by Trippoli et al. [8]. * The HTA reports for these five devices are available at the following web addresses: (1) http://www301.regione.toscana.it/bancadati/atti/Contenuto.xml?id=5272546&nomeFile=Decreto_n.19274_del_24-11-2020-Allegato-3; (2) http://www301.regione.toscana.it/bancadati/atti/Contenuto.xml?id=5318734&nomeFile=Decreto_n.2472_del_10-02-2022-Allegato-5; (3) http://www301.regione.toscana.it/bancadati/atti/Contenuto.xml?id=5245611&nomeFile=Decreto_n.3047_del_02-03-2020-Allegato-2; (4) http://www301.regione.toscana.it/bancadati/atti/Contenuto.xml?id=5310079&nomeFile=Decreto_n.20520_del_22-11-2021-Allegato-1; (5) http://www301.regione.toscana.it/bancadati/atti/Contenuto.xml?id=5255893&nomeFile=Decreto_n.9004_del_18-06-2020-Allegato-1. ^§^ Values in the Italian market were taken from the HTA reports. ^§§^ Values of QALYs were model-based in three cases out of five. ^†^ Survival at one year was 80.4% compared with 75.6% in controls (by considering patients of the International Registry of Acute Aortic Dissection (IRAD) [13] as controls); the survival gain is in life-years rather than QALYs. ^††^ Survival at one year was 36% compared with 23% in controls; the survival gain is in life-years rather than QALYs. ** Estimated according to Equation 7. CEA, cost-effectiveness analysis; HTA, health technology assessment; QALYs, quality-adjusted life years; VBP, value-based price; SOC, standard of care; GBP, Great Britain pound; R$, Brazilian real.

Device*	Unit price^§^	Reference (first author) suggested by CEA registry (c) or HTA report (h)	Treatment in the control group	Time horizon (months)		Gain in QALYs per patient^§§^	Value-based price**
1. Neovasc Reducer (EPS Vascular AB, Viken, Sweden): coronary sinus reducer stent	€6,500	Gallone et al. [11] (c,h)	Before and after comparison in included patients	12		0.138	VBP = €6,578. Parameters: othercosts_A_= €8,702, cost_B_ = €6,988, and gain_QALYs_ from column 5
2. Ascyrus Medical Dissection Stent, AMDS (CryoLife, Inc., Kennesaw, GA): hybrid aortic system for dissections	€13,000	Bozso et al. [12], Pape et al. [13] (h)	SOC	12		0.048^†^	VBP = €2,880. Parameters: othercosts_A_= €0, cost_B_ = €0, and gain_QALYs_ from column 5
3. Cardioband (Edwards Lifesciences, Irvine, CA): tricuspid valve reconstruction system	€22,000	Taramasso et al. [14] (h)	SOC		12	0.13^††^	VBP = €7,800. Parameters: othercosts_A_= €0, cost_B_ = €0, and gain_QALYs_ from column 5
4. Pascal Mitral Ace (Edwards Lifesciences, Irvine, CA): mitral valve transcatheter repair system	€22,000	Shore et al. [15] (h)	SOC		Lifetime	1.07	VBP = €45,272. Parameters: othercosts_A_ = GBP 26,471, cost_B_ = GBP 10,704, and gain_QALYs_ from column 5. Exchange rate: €1.00 = GPB 0.833
5. Cardia Ultrasept Dia (Cardia Inc., Eagan, MN): atrial septal defect closure device	€4,243	Costa et al. [16] (c)	Surgical closure		Lifetime	0.03	VBP = €3,579. Parameters: cost_B _= R$ 16,000, gain_QALYs_ from column 5, and othercosts_A_= R$ 6,836 (where R$ 6,836 is R$ 19,267 minus R$ 12,431; see [16] for further details). Exchange rate: €1.00 = R$ 5.15

**Figure 2:**
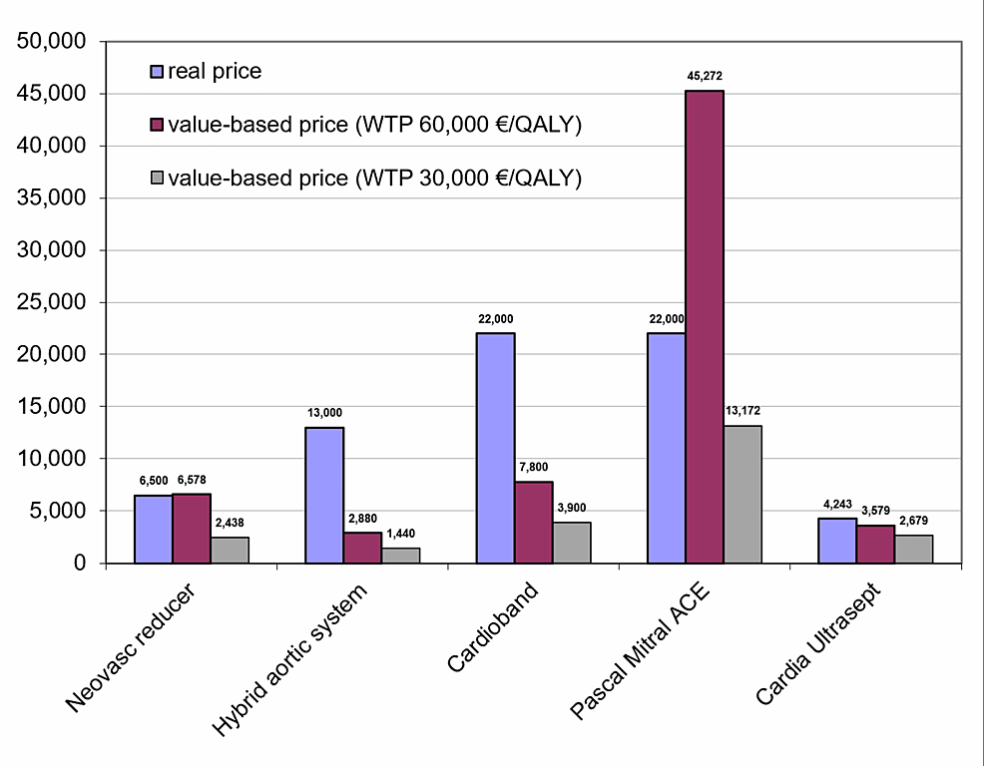
Comparison between value-based price and real price for the five devices included in the present analysis. In our primary analysis, the value-based price refers to the WTP threshold of €60,000 per QALY gained; in the secondary analysis, the threshold was €30,000 per QALY gained. Real prices are current prices from the Italian market. WTP, willingness to pay; QALY, quality-adjusted life year.

Using these cost-effectiveness data, we estimated the values of VBP shown in Table 1. For example, as regards the first device reported in Table 1 (Neovasc Reducer), the information requested by Equation 7 includes the following four parameters: WTP_threshold_, gain_QALYs_, cost_B_, and othercosts_A_. The WTP_threshold_ has the value of €60,000/QALY gained (or 30,000/QALY gained in the secondary analysis), gain_QALYs_ is 0.138 (drawn from Table 2 of [11]), cost_B _(i.e. the cost of the standard of care) is €6,988 (drawn from Table 2 of [11]), othercosts_A_ is €8,702 (calculated as the difference between €15,702 minus €7,000, where €15,702 is delta cost drawn from Table 2 of [11] and €7,000 is the cost of the device drawn from the paragraph “Healthcare resource use and costs” of [11]). The results of the consequent comparison between value-based price and real price for these five devices are shown in Figure 2.

In two cases, the value-based prices for the WTP threshold of €60,000/QALY were very similar to the real ones; in the other two cases, the real price was markedly higher than the value-based price. Finally, a value-based price higher than the real one was found for a device (Pascal Mitral Ace) for which the Markov model was extended over the lifetime horizon.

The figure reports also the value-based prices calculated in our secondary analysis according to the WTP threshold of €30,000/QALY. In this way, a range of prices is identified for each device. As a rule, the lower WTP threshold is more suitable for devices of wide use.

## Discussion

While in the field of medicines, specific institutions (such as national drug agencies) are responsible for the assessment of clinical evidence and for price negotiations required for reimbursement [4,17], in the field of devices, these two fundamental activities are not generally assigned to any such agencies but are typically managed by local institutions, in some cases at the regional level, but more frequently at sub-regional levels (e.g. local healthcare institutions or even individual hospitals) [18,19]. This determines great differences in how devices are managed not only among different countries but also within the same country [19]. This heterogeneity is the main reason that has prevented, in the field of devices, the development of standardised methods for the assessment of clinical evidence and for the application of cost-effectiveness principles (including the determination of value-based prices). While these considerations mainly refer to European countries, the overall picture is even more complex when other important countries are considered. For example, the United States represent a separate case for a variety of reasons (e.g. high expenditure for health care, lack of a national healthcare system, and presence of the Food and Drug Administration that approves both medicines and devices based on clinical characteristics) [4,18,19].

To our knowledge, the experience described in this paper is the first in which an institution of the healthcare system has evaluated a systematic application of VBP in the field of devices. The main areas in which VPB deserves to be applied include innovative devices, where innovation can be managed according to recent definitions [20], and include tenders for the procurement of high-technology devices [21], where VPB can be useful to determine the starting price of lots (the so-called auction base). On the one hand, innovative devices represent an area where price determination has relevant implications because the first price recognised for a new device has a long-lasting impact on the healthcare system. On the other hand, as regards tenders and their role in the procurement of devices, the values of starting price for the various lots are determined empirically, mainly on the basis of historical prices, and so introducing a pricing criterion based on the clinical benefit would represent an important advancement. One should keep in mind that the expenditure for some devices (particularly in very specialised medical disciplines such as cardiology [22]) can exceed the reimbursement derived from tariffs. This depends on the fact that some devices are priced at a higher level than their value-based price. Hence, the adoption of VBP could contribute to improving the current situation with a favourable impact in clinical and economic terms.

The results of our analysis raise two main comments and interpretations:

Point 1: When the literature search of cost-effectiveness data is successful, the estimation of value-based price is a quite straightforward task; unfortunately, the success rate for this search of data (21%) was relatively low in this pilot experience.

Point 2: It should be stressed that the method of VBP proposed herein is an approximate one. For example, when the reference cost-effectiveness study has been conducted in a foreign country, the various items of healthcare costs can be accepted as such in some cases (apart from the conversion from one currency into another), but in other cases adopting these estimates without further adaptations cannot be recommended.

Furthermore, the secondary analysis in which the WTP threshold of €30,000/QALY was adopted proved to be useful because a range could be proposed to determine the price level for each device, which is better than considering the single value of the highest acceptable price.

Healthcare costs (and particularly in-hospital costs) are known to have an acceptable degree of homogeneity across European countries and Canada; in these cases, differences in costs resulting from different currencies can be adequately managed by the simple conversion of one currency into another. In contrast, countries like the USA, Japan, Taiwan, Brazil, etc. (see [4]) present very remarkable intrinsic differences in their healthcare systems, and so the consequent differences in healthcare costs cannot be corrected by simple currency conversions. For example, the USA is characterised by two-fold or three-fold healthcare costs compared with European countries, particularly as regards in-hospital costs. Compared with the mere application of exchange rates, the method of purchasing power parity (PPP) represents a better tool for converting the price of any product from one currency into another [23], and can also be applied to medical products. In the context of our analysis, the application of PPP could have been useful, but we limited these conversions to simple exchange rates because the conversions based on PPP are typically focused on US dollars whereas our interest was focused on the European context.

All in all, when the available data allow for the estimation of value-based prices, the practical relevance of these estimates is extremely high, mainly because its absence would imply that devices are purchased at prices set by the manufacturer without any objective criterion.

As regards the limitations of the present work, the inability to determine the value-based price in 19 cases out of 24 represents a clear demonstration that the approach for VBP proposed herein does not presently have wide applicability. Furthermore, the drawbacks in cases where cost data were transferred from one country to another should not be overlooked, and so specific remedies will need to be devised and tested against real examples to improve the management of this issue.

Finally, knowing that the success rate of this preliminary application of VPB was low is an interesting finding because, given the undisputed usefulness of VBP for devices, this information of limited applicability will hopefully promote further cost-effectiveness studies investigating costs, clinical effectiveness, and utility.

## Conclusions

In conclusion, our preliminary results are encouraging and suggest a wider application of VBP in the field of high-technology medical devices. In this pilot experience, the availability of adequate literature data was a critical issue influencing the successful application of VBP. When adequate data are not available, how to fill this gap in the information remains an open question on which further debate will be needed.

## Appendices

Data availability

A simple computational tool incorporating Equation 7 is available at the following internet address: http://www.multicentredatabase.net/valuebasedprice2022.php.
